# Impressive Response with Brexpiprazole in Ekbom’s syndrome

**DOI:** 10.1192/j.eurpsy.2022.2054

**Published:** 2022-09-01

**Authors:** J. Hsiao, Y.-C. Shen

**Affiliations:** Hualien Tzu Chi Hospital, Department Of Psychiatry, No., Sec., Zhongyang Rd. Hualien City, Hualien County, Taiwan, Taiwan

**Keywords:** Antipsychotics, Brexpiprazole, Delusional parasitosis, Ekbom’s syndrome

## Abstract

**Introduction:**

Antipsychotics are the mainstream treatment of delusional disorder. However, limited therapeutic effect was recognized due to side effect and lack of insight.

**Objectives:**

This article presents a case with Ekbom’s syndrome, also known as delusional parasitosis, who has significant response with Brexpiprazole.

**Methods:**

A 58-year-old female developed her very first episode of psychosis 3 weeks before she visited our emergency department. Delusion of spiders laying eggs and bitsy spiders crawling over her body was claimed, followed by depressed mood and insomnia. The patient denied any substance use in recent months. Examination including biochemical studies, complete blood count, vitamin, and endocrine during admission were all normal. Brain image revealed senile cortical atrophy without apparent acute infarction. Cognitive abilities screening instrument (CASI) revealed total score 75, indicating borderline cognitive function. Ophthalmologist and dermatologist were consulted, and no specific abnormality was found.

**Results:**

Brexpiprazole 2mg was prescribed. After 3 weeks of treatment, the delusion improved with less parasitosis content. We discharged the patient, and kept following her at outpatient department with Brexpiprazole 2mg for 2 months. We tried to taper Brexpiprazole to 1mg at clinic, but her delusional parasitosis relapsed within 1 month. Therefore, we titrated the medication back to 2mg, and kept some dosage for 4 months. No more relapse of psychosis or significant movement dysfunction was observed. The total treatment course was 7 months.

**Conclusions:**

Brexpiprazole, with its D2 partial agonism, shows impressive antipsychotic effect to Ekbom’s syndrome. Little side effect was observed in clinical practice, making Brexpiprazole a worth-trying psychopharmacological management of delusional parasitosis.

**Disclosure:**

No significant relationships.

